# Miliary tuberculosis complicated with acute respiratory distress syndrome and hemophagocytic syndrome

**DOI:** 10.1093/omcr/omab142

**Published:** 2022-01-24

**Authors:** Kanako Takahashi, Masayuki Akatsuka, Shuji Yamamoto

**Affiliations:** Department of Anesthesiology, Obihiro Kosei Hospital, Obihiro, Hokkaido, Japan; Department of Anesthesiology, Obihiro Kosei Hospital, Obihiro, Hokkaido, Japan; Department of Anesthesiology, Obihiro Kosei Hospital, Obihiro, Hokkaido, Japan

**Figure 1:**
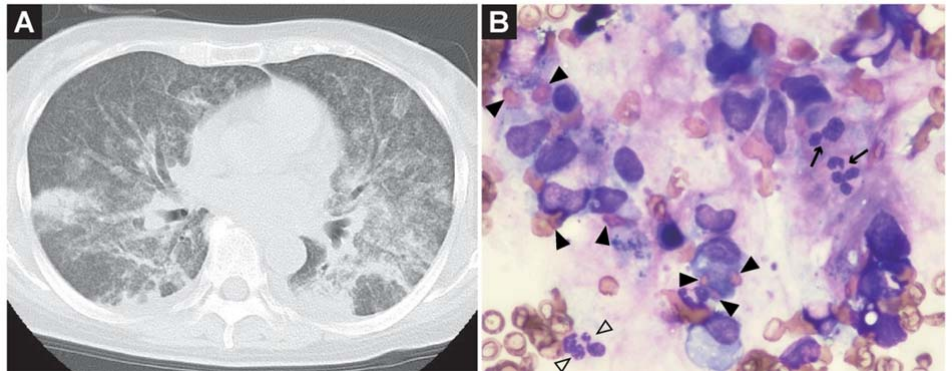
(**A**) CT images of the chest. Innumerable tiny, well-defined and miliary nodules are seen throughout the lungs and pleural surfaces; ground-glass opacification has spread to the bilateral lungs, reflecting overall air reduction in the lungs; (**B**) Images of bone marrow aspiration (Wright-Giemsa stain). Neutrophils with low granules and pseudo-Pergel nuclear abnormalities are noted; there is no increase in blast cells and no marked atypia of erythroblastic or megakaryocytic lineage; there are scattered phagocytic images throughout the specimen; the black triangles show the hemophagocytic image; the black triangles with white inside show the neutrophils with low granules, and the black arrows show the neutrophils with pseudo-Pergel nuclear abnormalities.

A 65-year-old woman presented to the emergency room with a 1-month history of fever and cough with expectoration. Chest radiography and computed tomography (CT) revealed miliary tuberculosis. A sputum sample was smear-positive for acid-fast bacilli and blood culture grew *Mycobacterium tuberculosis*. Rifampicin, isoniazid and streptomycin therapy was initiated. Four days after the initiation of therapy, her respiratory condition worsened: tachypnoea and tachycardia were observed, and mechanical ventilation with intubation was initiated. Meanwhile, according to the Berlin definition, she developed moderate acute respiratory distress syndrome (Fig. 1A). Her symptoms comprised septic shock; disseminated intravascular coagulation and deep venous thrombosis in the right posterior tibial and left peroneal veins, splenic and portal vein thrombosis and hemophagocytic syndrome (HPS) (Fig. 1B). Therefore, additional antimicrobial agents, recombinant thrombomodulin, antithrombin III and heparin were administered, and steroid was administered for HPS. The patient’s symptoms improved. She was extubated 5 days after admission and was transferred to a tuberculosis-designated medical institution uneventfully.

Miliary tuberculosis occurs in around 2% of all patients with tuberculosis, with a mortality rate of ~20% [1, 2]. Acute respiratory distress syndrome is noted in 7% of miliary tuberculosis cases. Moreover, miliary tuberculosis accounts for 2% of all cases of acute respiratory distress syndrome [3]. Herein, the patient developed miliary tuberculosis-related HPS. HPS can be classified into primary or secondary. Secondary HPS, which is associated with infection, is often caused by viral infection. Of note, *M. tuberculosis* has rarely been reported to cause bacterial HPS [4]. We suggest that the characteristic imaging findings in this case might help clinicians in establishing early diagnosis and treatment of affected patients.
